# Supplementary material for: Borrelia burgdorferi loses essential genetic elements and cell proliferative potential during stationary phase in culture but not in the tick vector
Source: bioRxiv. 2024 Oct 28:2024.10.28.620338. Preprint. [Version 1] doi: 10.1101/2024.10.28.620338 (PMC11565743; doi:10.1101/2024.10.28.620338)
Supplement: 3 [file NIHPP2024.10.28.620338v1-supplement-3.pdf]

bioRxiv preprint doi: https://doi.org/10.1101/2024.10.28.620338; this version posted October 28, 2024. The copyright holder for this preprint (which was not certified by peer review) is the author/funder, who has granted bioRxiv a license to display the preprint in perpetuity. It is made available under aCC-BY 4.0 International license.

**Figure S1. Stationary phase phenotypes characterized in various strains.**

Gray and white backgrounds indicate exponential and stationary phases, respectively.

**A.** Plot showing the percentage of cells (n = 72-417 for each strain and time points; see Supplemental File 1 for specific n values) from cultures used in Fig. 1A that remained negative for propidium iodide (PI) staining shown on a linear scale; these values were used to calculate the number of PI-negative cells. Results from two independent

bioRxiv preprint doi: https://doi.org/10.1101/2024.10.28.620338; this version posted October 28, 2024. The copyright holder for this preprint (which was not certified by peer review) is the author/funder, who has granted bioRxiv a license to display the preprint in perpetuity. It is made available under aCC-BY 4.0 International license.

cultures (biological replicates rep 1 and 2, represented by dark and light blue squares) of strain B31-MI are shown.

**B.** Plot showing the plating efficiency for B31-MI. Plating efficiency was calculated by dividing the CFU/mL of a culture by the number of cells/mL measured at each time point (shown in Fig. 1A). Asterisks indicate that the plating efficiency could not be plotted on a log scale, as the value was zero. Results from two independent cultures (biological replicates rep 1 and 2, represented dark and light pink triangles) of strain B31-MI are shown.

**C.** Plot showing culture densities (cells/mL, black circles) in comparison to colony-forming ability (CFU/mL, pink triangles) for a single culture of the clonal B31-derived strain S9.

**D.** Same as in (C) for clonal B31-derived strain CJW_Bb378.

**E.** Same as in (C) but for clonal B31-derived strain CJW_Bb379. Here, the density of viable cells in the culture was also determined by a liquid culture using microtiter plate-based limiting dilution assay. This density of viable cells is expressed as tissue culture infectious dose 50 ($TCID_{50}$) per mL (shown as orange hexagons).

**F.** Same as in Fig. 1A but for two independent cultures (biological replicates, rep 1 and 2) of strains 297 (left) and N40 (right). Black and grey circles represent culture density, dark and light blue squares represent PI-negative cells, and dark and light pink triangles represent CFU/mL for rep 1 and rep 2, respectively. Asterisks indicate that no colonies were detected.

**G.** Plots showing the percentage of PI-negative cells in the same cultures as used in (F) shown on a linear scale.

bioRxiv preprint doi: https://doi.org/10.1101/2024.10.28.620338; this version posted October 28, 2024. The copyright holder for this preprint (which was not certified by peer review) is the author/funder, who has granted bioRxiv a license to display the preprint in perpetuity. It is made available under aCC-BY 4.0 International license.

**Figure S2. Medium acidification in cultures and its effect on membrane permeability**

**A.** Plots showing the changes in pH that occurred during cultivation of strains B31-MI, N40, and 297 in BSK-II medium. These pH measurements were done using the same cultures as those for Figs. 1 and S1F-G. Gray and white backgrounds indicate exponential and stationary phases, respectively. Shown are results from two independent cultures (biological replicates, rep 1 and 2) of each indicated strain.

**B.** Plot showing the percentage of K2 cells that remained negative for propidium iodide (PI) uptake after culture at pH 6.0. There are the same results as in Fig. 2B, except on a linear scale.

**Figure S3. Effects of starvation in RPMI 1640 on various *B. burgdorferi* strains.** Results from two independent cultures (biological replicates, rep 1 and 2) of each respective strain are shown. See Supplemental File 1 for specific n values for each time point and strain.

**A.** Plots showing the percentage of cells from cultures of strain K2 (as used in Fig. 3), strain Bb914 (a derivative of strain 297), and non-clonal strain N40 that remained negative for propidium iodide (PI) uptake shown on a linear scale. For PI-negative cell determination, 48 to 311 cells were analyzed for each strain and time point.

**B.** Same as Fig. 3C for cultures of strains Bb914 and N40. For round-body determinations, 70 to 311 cells were analyzed for each strain and time point.

bioRxiv preprint doi: https://doi.org/10.1101/2024.10.28.620338; this version posted October 28, 2024. The copyright holder for this preprint (which was not certified by peer review) is the author/funder, who has granted bioRxiv a license to display the preprint in perpetuity. It is made available under aCC-BY 4.0 International license.

**C.** Same as Fig. 3D for cultures of Bb914 and N40. For PI-negative cell determination, 70 to 311 cells were analyzed for each strain and time point.

**Figure S4. Changes to DNA staining patterns in stationary phase cells.**

For panels B-G, gray and white backgrounds indicate exponential and stationary phases, respectively. Results from two independent cultures (biological replicates, rep 1 and 2) of each respective strain are shown. See Supplemental File 1 for specific n values for each time point and strain.

**A.** Representative images of a cell from an exponential or stationary phase culture of B31-MI where DNA was visualized by staining with Hoechst 33342. Light blue arrowheads indicate gaps depleted of DNA signal in the stationary phase cell.

**B.** Plot showing the percentage of the cell populations with one continuous DNA signal for the B31-MI cultures used in Fig. 1. For DNA object detection analysis, 72 to 418 cells were analyzed for each strain and time point.

**C.** Same as in (B) except for cultures of strain 297, which were the same cultures as those used for Fig. S1F. For DNA object detection analysis, 66 to 332 cells were analyzed for each strain and time point.

**D.** Same as in (B) except for cultures of strain N40, which were the same cultures as those used for Fig. S1F. For DNA object detection analysis, 45 to 350 cells were analyzed for each strain and time point.

**E.** Plot showing the decay of the Hoechst signal intensity in BM31-MI cells as a function of culture age. Mean whole cell intensity of either biological replicate for each time point

bioRxiv preprint doi: https://doi.org/10.1101/2024.10.28.620338; this version posted October 28, 2024. The copyright holder for this preprint (which was not certified by peer review) is the author/funder, who has granted bioRxiv a license to display the preprint in perpetuity. It is made available under aCC-BY 4.0 International license.

was fit to an exponential decay to illustrate the sharp decrease in signal. The same B31-MI culture was used as in Fig. 1. A.U. indicates arbitrary units.

**F.** Same as in (E) except for cultures of strain 297.

**G.** Same as in (E) except for cultures of strain N40.

**Figure S5. Decrease in *oriC* and plasmid copy density during stationary phase.**

**A.** Plot showing changes in *oriC* copies per cell in cultures of strains CJW_Bb378 and CJW_Bb379 (both clonal B31 derivatives) in BSK-II medium. The numbers of *oriC* copies were determined from fluorescence microscopy images by counting the fluorescent foci of mCherry-ParB (CJW_Bb379) or ParZ-GFP (CJW_Bb378) that indicate the subcellular location of the endogenous *parAZBS* region adjacent to *oriC* (52). One culture for each strain was analyzed at the indicated time points. Shown are means ± standard deviations across cells. For each strain and time point, 60 to 387 cells were analyzed (see Supplemental File 1 for specific n values). Gray and white backgrounds indicate exponential and stationary phases, respectively.

**B.** Same as in (A) except that the mean *oriC* densities (expressed as *oriC* copies per 10 µm of cell length) are plotted.

**C.** Plot showing plasmid densities (expressed as plasmid copy number per 10 µm of cell) in exponential phase (grey bars) and after two days in stationary phase (teal bars) using the same cultures as in Fig. 4C. Each black dot represents an independent biological replicate. Only cells (n = 22-167) with at least one clear *oriC* focus were considered in this analysis. The strain identities and the number of cells analyzed for each data point are detailed in Supplemental File 1.

bioRxiv preprint doi: https://doi.org/10.1101/2024.10.28.620338; this version posted October 28, 2024. The copyright holder for this preprint (which was not certified by peer review) is the author/funder, who has granted bioRxiv a license to display the preprint in perpetuity. It is made available under aCC-BY 4.0 International license.

**D.** Plot showing the intensity profiles for mCherry-ParB and GFP-ParB$^{P1}$ signals along the cell length for the CJW_Bb489 cell shown in Fig. 4E. The division site, reflected by the dip in fluorescence signal, is indicated by red arrowheads. A.U. stands for arbitrary units.

**E.** Representative phase contrast and fluorescence images of cells of strain CJW_Bb203 in which cp26 is labeled with msfGFP-ParB$^{P1}$. Cells were from a population in exponential phase or in stationary phase for two days. Yellow arrowheads point to a stationary phase cell with clear fluorescent cp26 foci.

**Figure S6. Assessment of the plasmid content in *B. burgdorferi* clones isolated after growth in laboratory cultures.**

Multiplex PCR was done using plasmid-specific primer pairs previously validated for use on strain B31 (71) and grouped in six sets, as shown in the images. The PCR products were separated by electrophoresis and visualized by SYBR Safe staining and automated detection using a Bio-Rad ChemiDoc Imaging System gel imager. The intensity of the resulting images was scaled to allow for visual detection of the weakly positive bands. As a result of acquisition and scaling, some of the more intense bands are saturated. All multiplex PCR results for all clones analyzed are summarized in Table 1.

**A**. Gel images for PCR products obtained by multiplex PCR profiling of all clones tested from 10-day-old stationary phase culture of CJW_Bb523. Lost plasmids (written in red) are indicated with a red asterisk on the gel image. Each clone tested is given a unique identifier: first A or B, corresponding to biological replicate 1 or 2, then S for stationary

bioRxiv preprint doi: https://doi.org/10.1101/2024.10.28.620338; this version posted October 28, 2024. The copyright holder for this preprint (which was not certified by peer review) is the author/funder, who has granted bioRxiv a license to display the preprint in perpetuity. It is made available under aCC-BY 4.0 International license.

phase, followed by the identification number of the screened colony (see Table 1 for details).

**B.** Same as in (A) except that from cultures in exponential phase. Each clone tested is given a unique identifier as described in (A) except that E is for exponential phase. The (+) indicates a positive control sample, which corresponds to strain CJW_Bb523 isolated in exponential phase.

**Figure S7. Assessment of the plasmid content in *B. burgdorferi* clones isolated from unfed ticks.**

Same as in Fig. S6 except that the clones were isolated from the unfed ticks used for Fig. 5. Shown are gel images of PCR products from clones obtained by plating crushed nymphs colonized with strain CJW_Bb474 and maintained unfed at room temperature for 14 months after molt. CJW_Bb474 is a clonal B31-MI derivative that lacks lp5, cp9, and lp56. Isolated and tested clones are grouped by nymph.

**Figure S8. Determination of cell proliferative potential and *oriC* copy number per cell for cultures grown at room temperature.**

Gray and white backgrounds indicate exponential and stationary phases, respectively. Results from two independent cultures (biological replicates, rep 1 and 2) of strain CJW_Bb379 are shown. See Supplemental File 1 for specific n values for each time point.

**A.** Plot showing culture densities (cells/mL, black and gray circles) in comparison to colony forming ability (CFU/mL, dark and light pink triangles) for two cultures of

bioRxiv preprint doi: https://doi.org/10.1101/2024.10.28.620338; this version posted October 28, 2024. The copyright holder for this preprint (which was not certified by peer review) is the author/funder, who has granted bioRxiv a license to display the preprint in perpetuity. It is made available under aCC-BY 4.0 International license.

CJW_Bb379 grown in BSK-II at room temperature (~21°C). Due to the slower growth rate associated with the lower temperature, the x-axis is standardized relative to weeks in stationary phase.

**B.** Plot showing changes in *oriC* copies per cell over time for the two cultures of CJW_Bb379 grown in BSK-II at room temperature (~21°C) using the cultures from (A). For *oriC* copy number quantification, 7 to 811 cells were analyzed for each strain and time point.

bioRxiv preprint doi: https://doi.org/10.1101/2024.10.28.620338; this version posted October 28, 2024. The copyright holder for this preprint (which was not certified by peer review) is the author/funder, who has granted bioRxiv a license to display the preprint in perpetuity. It is made available under aCC-BY 4.0 International license.

# Figure S1

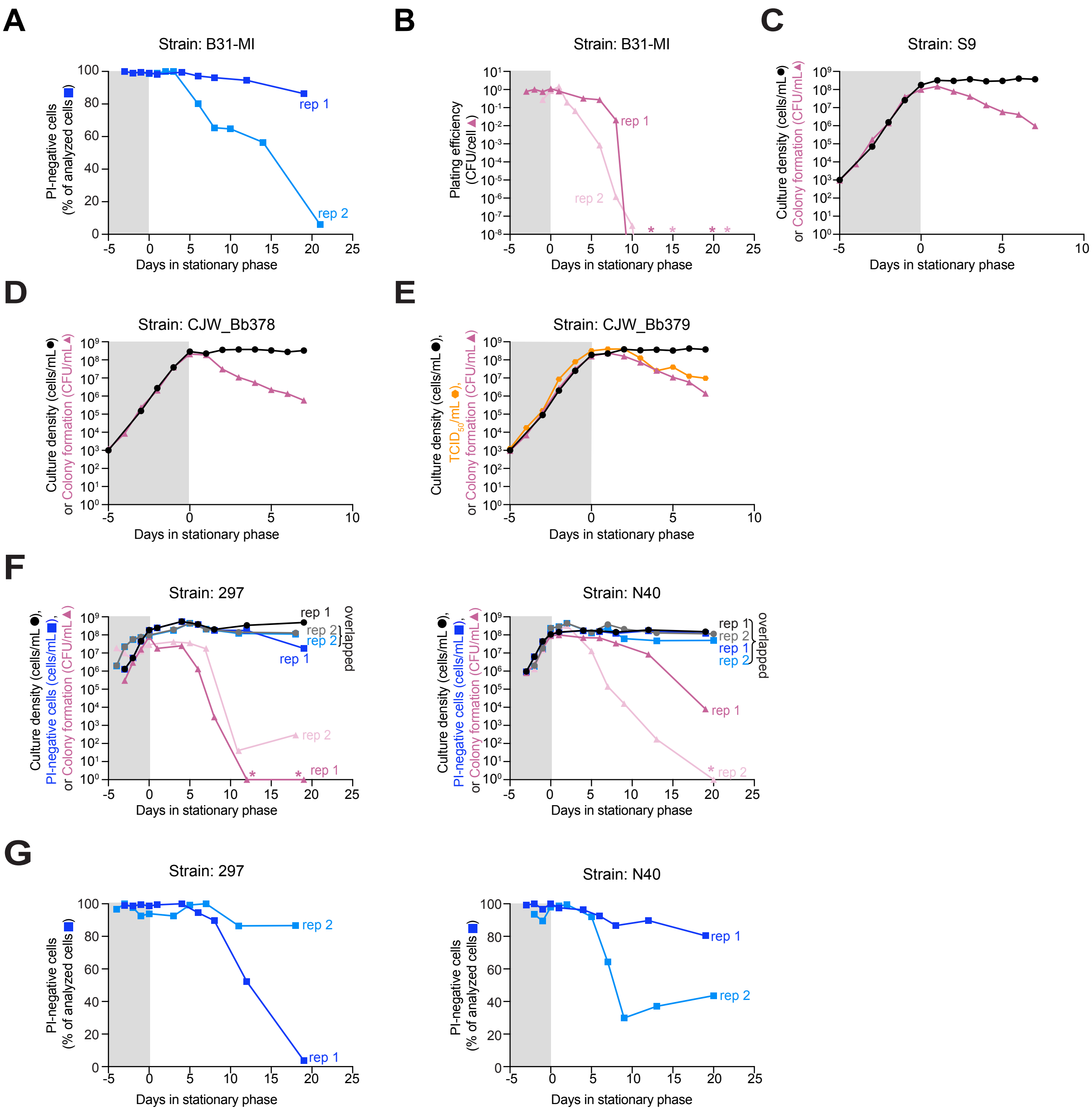

bioRxiv preprint doi: https://doi.org/10.1101/2024.10.28.620338; this version posted October 28, 2024. The copyright holder for this preprint (which was not certified by peer review) is the author/funder, who has granted bioRxiv a license to display the preprint in perpetuity. It is made available under aCC-BY 4.0 International license.

# Figure S2

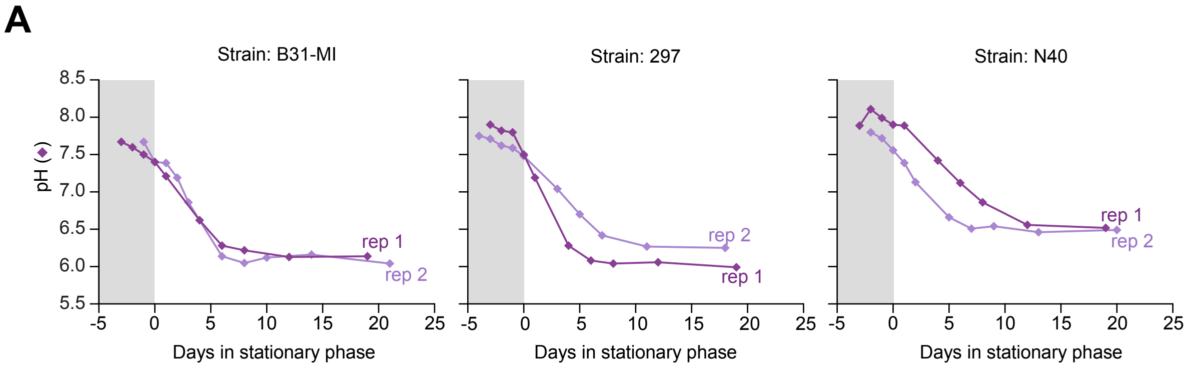


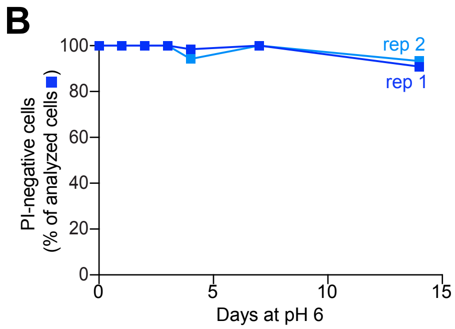

bioRxiv preprint doi: https://doi.org/10.1101/2024.10.28.620338; this version posted October 28, 2024. The copyright holder for this preprint (which was not certified by peer review) is the author/funder, who has granted bioRxiv a license to display the preprint in perpetuity. It is made available under aCC-BY 4.0 International license.

# Figure S3

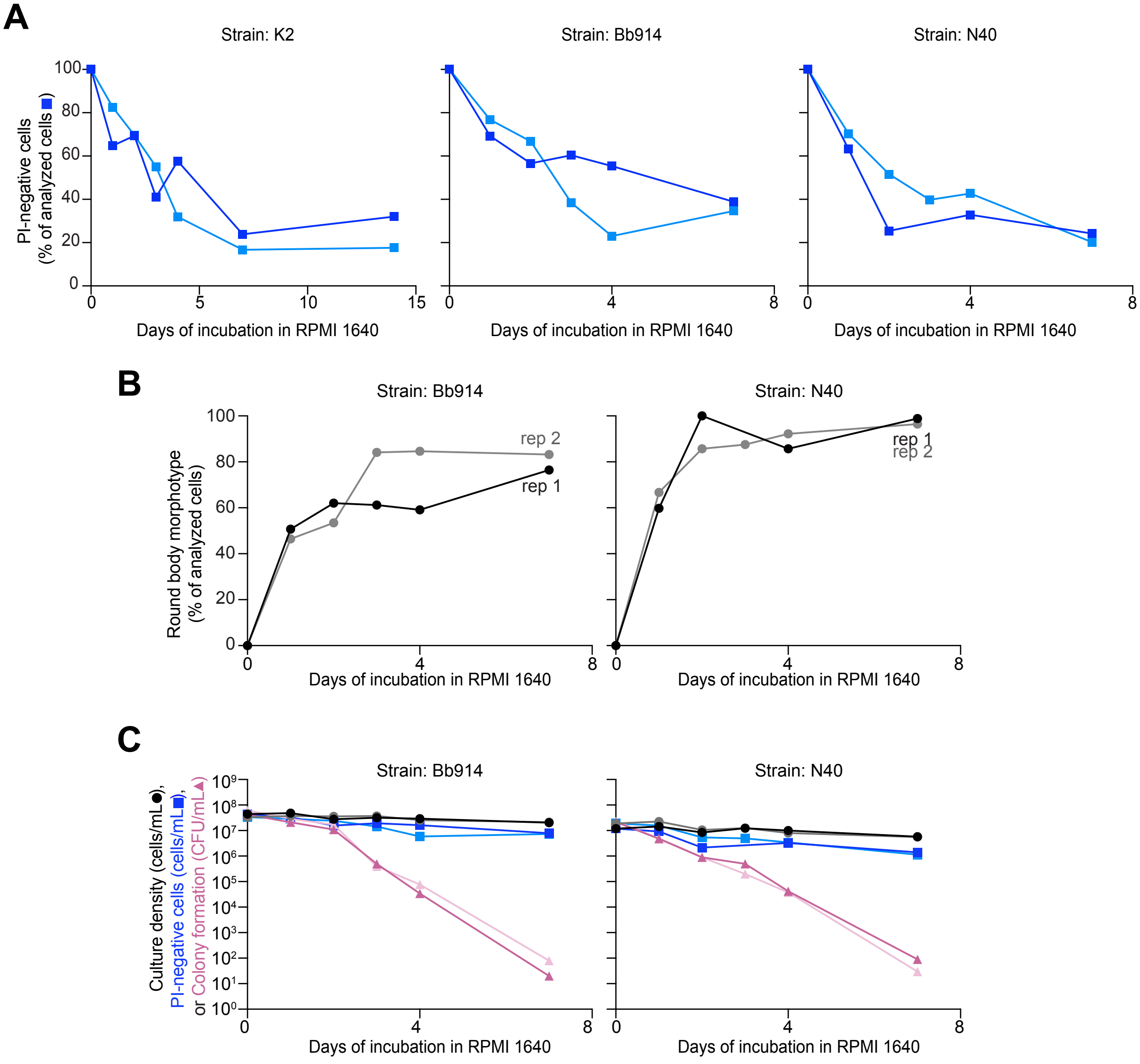

bioRxiv preprint doi: https://doi.org/10.1101/2024.10.28.620338; this version posted October 28, 2024. The copyright holder for this preprint (which was not certified by peer review) is the author/funder, who has granted bioRxiv a license to display the preprint in perpetuity. It is made available under aCC-BY 4.0 International license.

# Figure S4

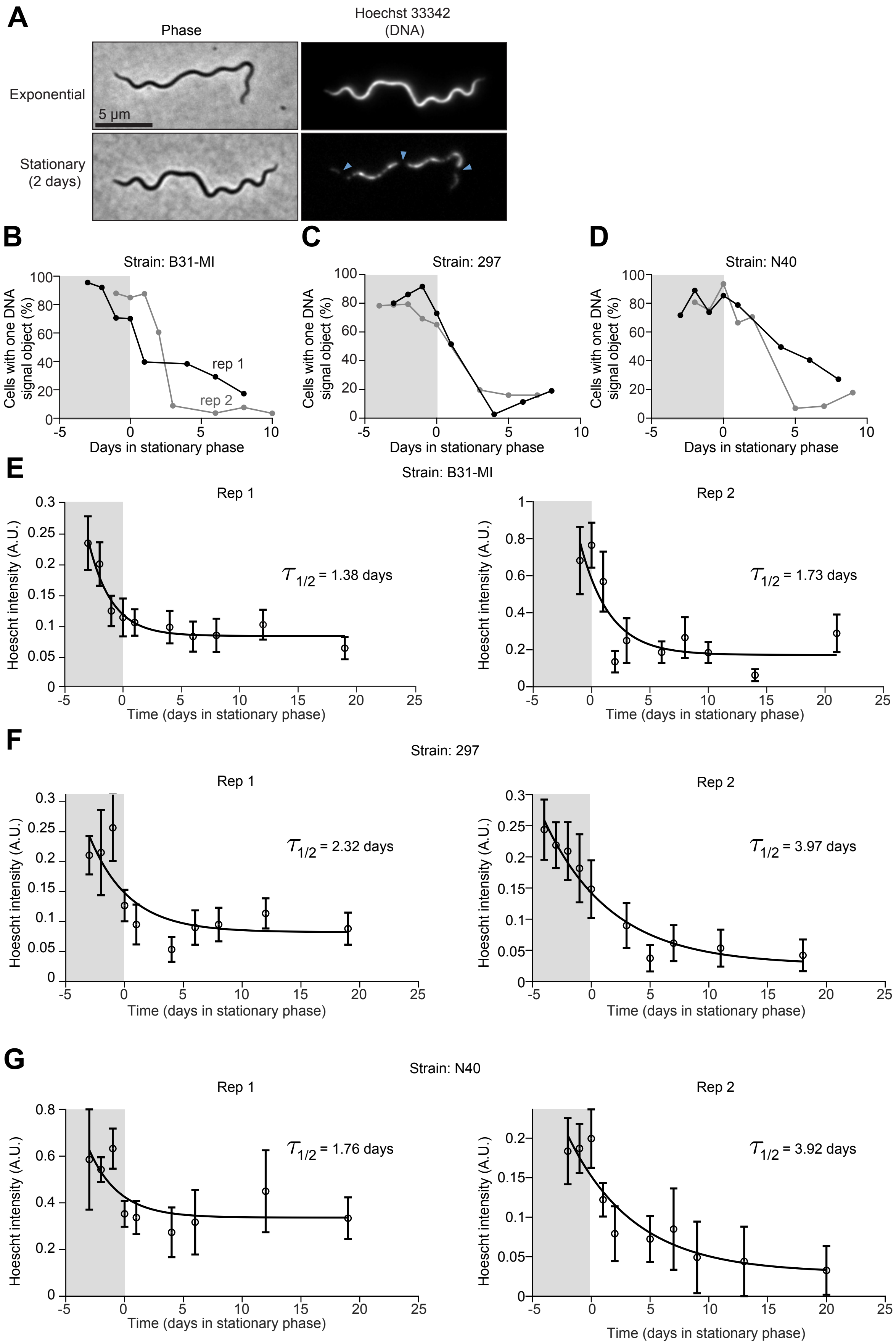

bioRxiv preprint doi: https://doi.org/10.1101/2024.10.28.620338; this version posted October 28, 2024. The copyright holder for this preprint (which was not certified by peer review) is the author/funder, who has granted bioRxiv a license to display the preprint in perpetuity. It is made available under aCC-BY 4.0 International license.

# Figure S5

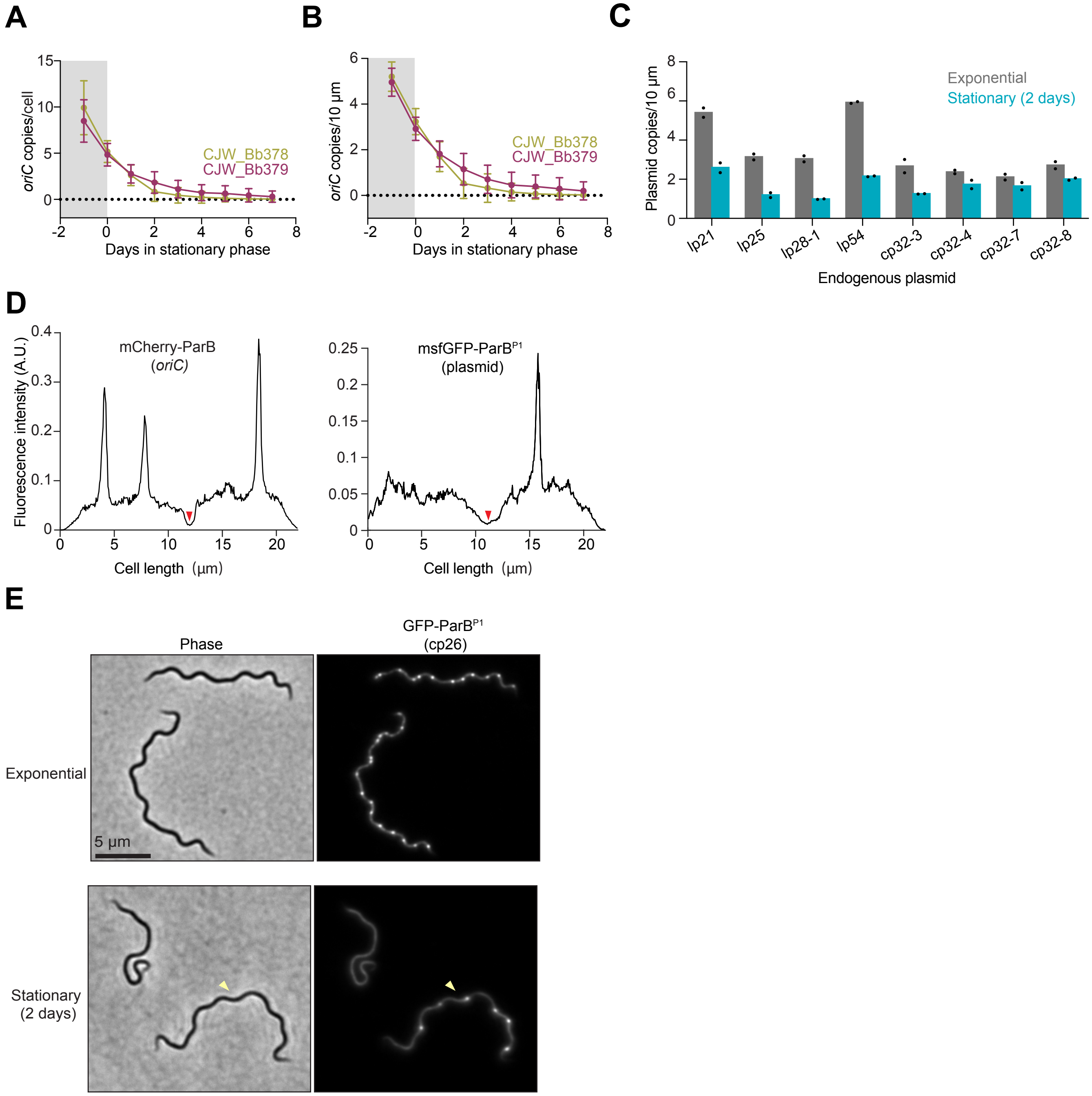

bioRxiv preprint doi: https://doi.org/10.1101/2024.10.28.620338; this version posted October 28, 2024. The copyright holder for this preprint (which was not certified by peer review) is the author/funder, who has granted bioRxiv a license to display the preprint in perpetuity. It is made available under aCC-BY 4.0 International license.

# Figure S6

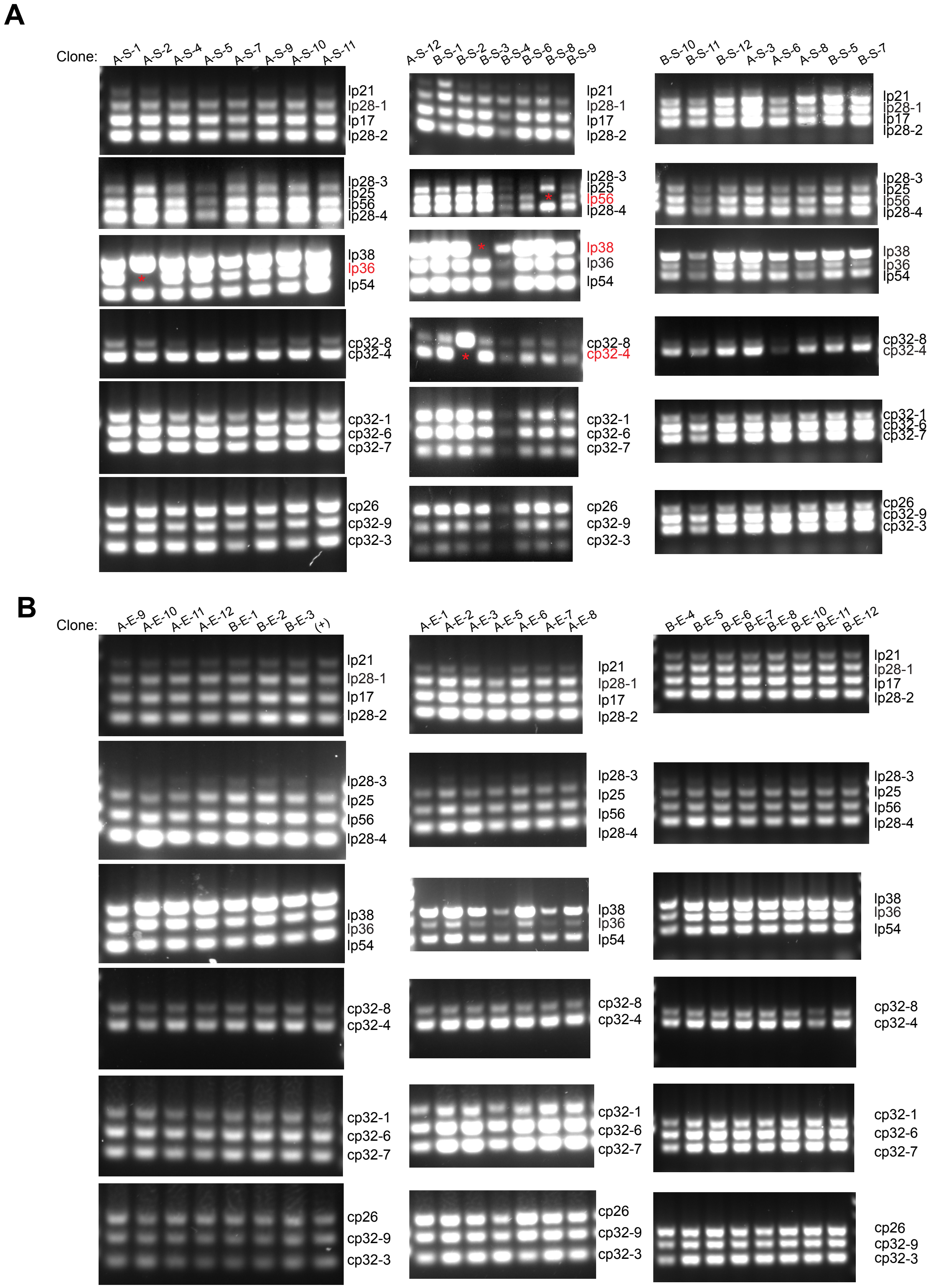

bioRxiv preprint doi: https://doi.org/10.1101/2024.10.28.620338; this version posted October 28, 2024. The copyright holder for this preprint (which was not certified by peer review) is the author/funder, who has granted bioRxiv a license to display the preprint in perpetuity. It is made available under aCC-BY 4.0 International license.

# Figure S7

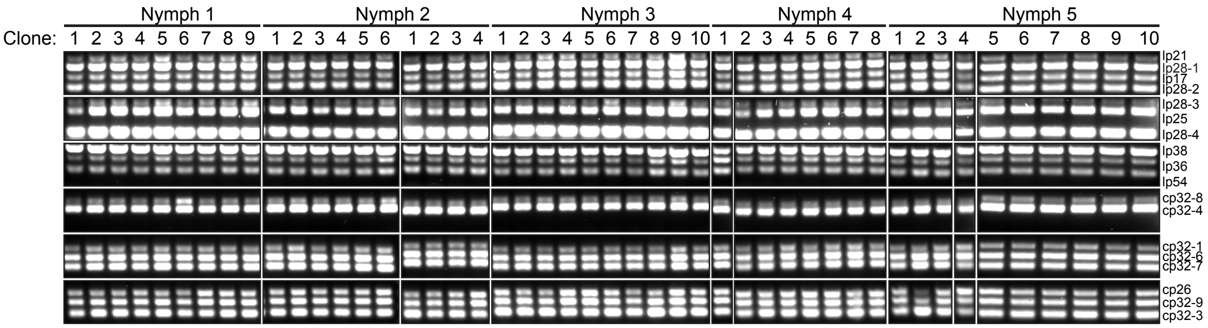

bioRxiv preprint doi: https://doi.org/10.1101/2024.10.28.620338; this version posted October 28, 2024. The copyright holder for this preprint (which was not certified by peer review) is the author/funder, who has granted bioRxiv a license to display the preprint in perpetuity. It is made available under aCC-BY 4.0 International license.

# Figure S8

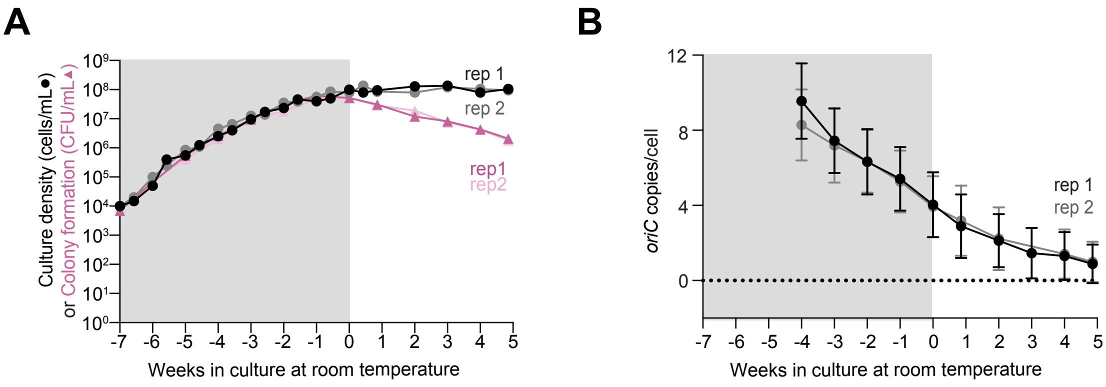

bioRxiv preprint doi: https://doi.org/10.1101/2024.10.28.620338; this version posted October 28, 2024. The copyright holder for this preprint (which was not certified by peer review) is the author/funder, who has granted bioRxiv a license to display the preprint in perpetuity. It is made available under aCC-BY 4.0 International license.

**Table S1. List of strains used in this study.**

| **Strain** | **Description** | **Reference** |
|---|---|---|
| B31-MI | Type strain (non-clonal) | (1–3) |
| N40 | Widely used strain (non-clonal) | (4, 5) |
| 297 | Widely used strain (non-clonal) | (6–9) |
| Bb914 | 297 clonal derivative that expresses cytosolic GFP | (10) |
| K2 | Transformable, infectious B31 clone | (11) |
| S9 | Transformable, infectious B31 clone | (11) |
| CJW_Bb203 | S9 derivative with fluorescently labeled cp26 copies and *oriC* copies | (12) |
| CJW_Bb274 | S9 derivative with fluorescently labeled lp25 and *oriC* copies | (12) |
| CJW_Bb326 | S9 derivative with fluorescently labeled lp54 and *oriC* copies | (12) |
| CJW_Bb378 | S9 derivative with *oriC* labeled by ParZ-GFP | (12) |
| CJW_Bb379 | S9 derivative with *oriC* labeled by mCherry-ParB | (12) |
| CJW_Bb474 | CJW_Bb379 derivative that expresses cytosolic GFP | (12) |
| CJW_Bb489 | S9 derivative with fluorescently labeled lp28-1 copies | (12) |
| CJW_Bb515 | B31 derivative with fluorescently labeled cp32-3 copies | (12) |
| CJW_Bb516 | B31 derivative with fluorescently labeled cp32-7 copies | (12) |
| CJW_Bb517 | B31 derivative with fluorescently labeled cp32-4 copies | (12) |
| CJW_Bb518 | B31 derivative with fluorescently labeled cp32-8 copies | (12) |
| CJW_Bb523 | B31 clone, lacks lp5 and cp9 | (12) |
| CJW_Bb526 | S9 derivative with fluorescently labeled lp21 copies | (12) |

bioRxiv preprint doi: https://doi.org/10.1101/2024.10.28.620338; this version posted October 28, 2024. The copyright holder for this preprint (which was not certified by peer review) is the author/funder, who has granted bioRxiv a license to display the preprint in perpetuity. It is made available under aCC-BY 4.0 International license.

## References

1. Burgdorfer W, Barbour AG, Hayes SF, Benach JL, Grunwaldt E, Davis JP. 1982. Lyme disease-a tick-borne spirochetosis? Science 216:1317–1319.

2. Fraser CM, Casjens S, Huang WM, Sutton GG, Clayton R, Lathigra R, White O, Ketchum KA, Dodson R, Hickey EK, Gwinn M, Dougherty B, Tomb JF, Fleischmann RD, Richardson D, Peterson J, Kerlavage AR, Quackenbush J, Salzberg S, Hanson M, van Vugt R, Palmer N, Adams MD, Gocayne J, Weidman J, Utterback T, Watthey L, McDonald L, Artiach P, Bowman C, Garland S, Fuji C, Cotton MD, Horst K, Roberts K, Hatch B, Smith HO, Venter JC. 1997. Genomic sequence of a Lyme disease spirochaete, Borrelia burgdorferi. Nature 390:580–586.

3. Casjens S, Palmer N, van Vugt R, Huang WM, Stevenson B, Rosa P, Lathigra R, Sutton G, Peterson J, Dodson RJ, Haft D, Hickey E, Gwinn M, White O, Fraser CM. 2000. A bacterial genome in flux: the twelve linear and nine circular extrachromosomal DNAs in an infectious isolate of the Lyme disease spirochete Borrelia burgdorferi. Mol Microbiol 35:490–516.

4. Barthold SW, Moody KD, Terwilliger GA, Duray PH, Jacoby RO, Steere AC. 1988. Experimental Lyme arthritis in rats infected with Borrelia burgdorferi. J Infect Dis 157:842–846.

5. Barthold SW, Moody KD, Terwilliger GA, Jacoby RO, Steere AC. 1988. An animal model for Lyme arthritis. Ann N Y Acad Sci 539:264–273.

bioRxiv preprint doi: https://doi.org/10.1101/2024.10.28.620338; this version posted October 28, 2024. The copyright holder for this preprint (which was not certified by peer review) is the author/funder, who has granted bioRxiv a license to display the preprint in perpetuity. It is made available under aCC-BY 4.0 International license.

6. Steere AC, Grodzicki RL, Kornblatt AN, Craft JE, Barbour AG, Burgdorfer W, Schmid GP, Johnson E, Malawista SE. 1983. The spirochetal etiology of Lyme disease. N Engl J Med 308:733–740.

7. Steere AC, Grodzicki RL, Craft JE, Shrestha M, Kornblatt AN, Malawista SE. 1984. Recovery of Lyme disease spirochetes from patients. Yale J Biol Med 57:557–560.

8. Schutzer SE, Fraser-Liggett CM, Casjens SR, Qiu W-G, Dunn JJ, Mongodin EF, Luft BJ. 2011. Whole-genome sequences of thirteen isolates of Borrelia burgdorferi. J Bacteriol 193:1018–1020.

9. Casjens SR, Mongodin EF, Qiu W-G, Luft BJ, Schutzer SE, Gilcrease EB, Huang WM, Vujadinovic M, Aron JK, Vargas LC, Freeman S, Radune D, Weidman JF, Dimitrov GI, Khouri HM, Sosa JE, Halpin RA, Dunn JJ, Fraser CM. 2012. Genome stability of Lyme disease spirochetes: comparative genomics of Borrelia burgdorferi plasmids. PLoS One 7:e33280.

10. Dunham-Ems SM, Caimano MJ, Pal U, Wolgemuth CW, Eggers CH, Balic A, Radolf JD. 2009. Live imaging reveals a biphasic mode of dissemination of Borrelia burgdorferi within ticks. J Clin Invest 119:3652–3665.

11. Rego ROM, Bestor A, Rosa PA. 2011. Defining the plasmid-borne restriction-modification systems of the Lyme disease spirochete Borrelia burgdorferi. J Bacteriol 193:1161–1171.

12. Takacs CN, Wachter J, Xiang Y, Ren Z, Karaboja X, Scott M, Stoner MR, Irnov I, Jannetty N, Rosa PA, Wang X, Jacobs-Wagner C. 2022. Polyploidy, regular

bioRxiv preprint doi: https://doi.org/10.1101/2024.10.28.620338; this version posted October 28, 2024. The copyright holder for this preprint (which was not certified by peer review) is the author/funder, who has granted bioRxiv a license to display the preprint in perpetuity. It is made available under aCC-BY 4.0 International license.

patterning of genome copies, and unusual control of DNA partitioning in the Lyme disease spirochete. Nat Commun 13:7173.
